# Supplementary material for: Machine Learning Approximations to Predict Epigenetic Age Acceleration in Stroke Patients
Source: Int J Mol Sci. 2023 Feb 1;24(3):2759. doi: 10.3390/ijms24032759 (PMC9917369; doi:10.3390/ijms24032759)
Supplement: Supplementary file 1 [file ijms-24-02759-s001.zip › ijms-2148790-supplementary.pdf]

Supplementary table S1: Missing data of each variable and imputed variables

	Missing data	Imputation
Age	0	-
Biological Age	0	-
Sex	0	-
Basal mRS	0	-
Smoking	10	Yes
Alcoholism	34	Yes
Drug consumption	30	Yes
Weight	57	Yes
Height	83	Yes
BMI	90	Yes
Waist	238	-
Hypertension	0	-
Diabetes	0	-
Hyperlipidemia	0	-
Ischemic heart disease	0	-
Atrial fibrillation	0	-
Heart ejection fraction	621	-
Moderate physical activity	897	-
Brain parenchymal fraction	740	-
Laboratory determinations:		
Leukocytes	18	Yes
Neutrophils	26	Yes
Lymphocytes	61	Yes
Monocytes	57	Yes
Total cholesterol	120	Yes
Triglycerides	123	Yes
HDL	177	Yes
LDL	174	Yes
HbA1c	480	-
Creatinine	239	-
Estimated glomerular filtration rate	242	-
C-reactive protein	284	-
Diet variables (daily intake):		
Energy	805	-
carbohydrates	805	-
proteins	805	-
Total fat	805	-
Saturated fat	805	-
Monounsaturated fat	805	-
Polyunsaturated fat	805	-
Cholesterol	805	-
Fiber	805	-
Coffee	235	-
Mediterranean score	900	-

The table shows missing data of all the variables registered from the 952 individuals, and the information about which variables were imputed.

Supplementary table S2: Hyperparameters tunned during the training phase for each model

Model	Hyperparameters	Best tunning
Elastic Net Regression	Alpha	0.184
	Lambda	0.776
K Nearest Neighbors	K	20
Random Forest	mtry	10
	Split rule	Variance
	Minimum node size	10
Support vector machine	Sigma	0.024
	C	0.25
Multilayer perceptron	Activation function	Identity
	Alpha	0.0001
	Hidden layers	(20, 10, 5)
	Learning rate	Adaptative
	Maximum iterations	10000
	Solver	SGD

The table shows which hyperparameters were tunned via cross validation for each model. We also show the best performing configuration in each case (lower root mean squared error).

Keyword: SGD, Stochastic Gradient Descent.

Supplementary table S3: Lineal regression model

	Beta value	Standard Error	t value	P value
Smoking	-0.73	0.61	-1.19	0.233
Alcoholism - Ex >1 year	-0.91	1.12	-0.81	0.418
Alcoholism - Yes	1.59	0.61	2.60	0.009
Age (C-Age)	-0.29	0.02	-12.47	2.28E-32
Sex - Male	1.09	0.63	1.72	0.085
Weight (kg)	0.02	0.02	0.85	0.397
Height (cm)	0.05	0.03	1.48	0.139
Drug consumption	-1.44	1.73	-0.83	0.404
Atrial fibrillation	0.55	0.53	1.05	0.294
Leukocytes (log)	0.82	1.17	0.70	0.486
Ischemic heart disease	-0.81	0.68	-1.18	0.239
Neutrophils (log)	1.75	0.88	2.00	0.046
Triglycerides (log)	0.037	0.60	0.06	0.950
HDL	-0.01	0.02	-0.74	0.456
Basal mRS	-0.08	0.21	-0.37	0.712

We show the association between Aging-A and each predictor in the linear regression model, including beta coefficient assigned to each one, standard error, t-value and p-value.

Supplementary table S4: Elastic net regression model

	Beta value
Age	-7.98E-02
Weight	
Height	1.50E-02
Leukocytes (log transformation)	2.16E-01
Neutrophils (log)	2.93E-01
Lymphocytes	
Monocytes (log)	2.70E-01
Total cholesterol	
Triglycerides (log)	1.13E-03
HDL	
LDL	
BMI (log)	
Age^2	-6.85E-04
Age^3	-7.22E-06
Weight^2 (log)	4.51E-01
Weight^3 (log)	2.93E-01
Height^2	3.51E-05
Heigth^3	1.09E-07
Leukocytes^2 (log)	9.61E-02
Leukocytes^3 (log)	5.72E-02
Neutrophils^2 (log)	1.46E-01
Neutrophils^3 (log)	9.60E-02
Lymphocytes^2 (log)	-2.84E-01
Lymphocytes^3 (log)	-1.81E-01
Monocytes^2 (log)	1.24E-01
Monocytes^3 (log)	7.04E-02
Total cholesterol^2 (log)	-2.58E-01
Total cholesterol^3 (log)	-1.56E-01
Triglycerides^2 (log)	
Triglycerides^3 (log)	
HDL^2 (log)	
HDL^3 (log)	
LDL^2 (log)	
LDL^3 (log)	
BMI^2 (log)	
BMI^3 (log)	
Smoking - Yes	
Alcoholism - Ex >1 year	-4.32E-01
Alcoholism - Yes	1.18
Sex - Male	6.19E-01
Basal mRS 1	
Basal mRS 2	
Basal mRS 3	
Basal mRS 4-5	
Drug consumption - Yes	
Hypertension - Yes	4.47E-01
Diabetes - Yes	
Hyperlipidemia - Yes	
Ischemic heart disease - Yes	-5.01E-01
Atrial fibrillation - Yes	

Beta values assigned to each predictor in the EN model after training.

Supplementary table S5: Associations between those variables excluded of the models (due to high proportion of missing cases) and predictions and residuals of best performing models.

		EN Predictions		EN Residuals		MLP Predictions		MLP Residuals		
	**Mean (±SD)/Median(IQR)**	**r**	***p*-value**	**r**	***p*-value**	**r**	***p*-value**	**r**	***p*-value**	**N**
Diet variables, daily intake:										
Energy, kilocalories	2580 (±1253)	0.161	0.0507	-0.035	0.6763	0.170	0.0397	-0.049	0.5565	147
carbohydrates, g	249 (±121)	0.099	0.2334	-0.010	0.9038	0.107	0.198	-0.021	0.8019	147
proteins, g	122 (±63)	0.081	0.3285	-0.039	0.6428	0.069	0.405	-0.033	0.6948	147
Total fat, g	120 (±67)	0.140	0.0906	-0.073	0.3803	0.154	0.0626	-0.091	0.2714	147
Saturated fat, g	35 (±20)	0.130	0.1158	-0.074	0.3707	0.142	0.0856	-0.091	0.2739	147
Monounsaturated fat, g	59(±33)	0.137	0.0989	-0.081	0.3294	0.153	0.0641	-0.102	0.2212	147
Polyunsaturated fat, g	18 (±12)	0.132	0.1107	-0.049	0.5587	0.141	0.0896	-0.062	0.4570	147
Cholesterol, g	456 (±305)	0.112	0.1774	0.026	0.7587	0.105	0.2073	0.027	0.7438	147
Fiber, g	29 (±16)	-0.011	0.8908	-0.015	0.8567	-0.007	0.933	-0.018	0.8248	147
Coffee, cups	1 (±1.2)	0.213	<0.0001	0.003	0.9273	0.195	<0.0001	0.006	0.8632	717
Mediterranean score	17,21 (±2,16)	-0.373	0.0065	-0.030	0.8301	-0.271	0.052	-0.071	0.6150	52
Moderate physical activity, min/month	600 (240-1590)	-0.279	0.0395	-0.003	0.9806	-0.189	0.1668	-0.041	0.7684	55
Waist, cm	99,5(±14,7)	0.172	<0.0001	0.025	0.5120	0.160	<0.0001	0.025	0.508	714
HbA1c, %	6,4 (±1,6)	0.077	0.0955	0.063	0.1708	0.075	0.1058	0.062	0.1791	472
Creatinine, mg/dL	1 (±0,58)	-0.007	0.8487	-0.010	0.7864	0.008	0.8253	-0.021	0.5832	713
Estimated glomerular filtration rate, CKD-EPI	73,29 (± 22,55)	0.442	<0.0001	0.060	0.1073	0.422	<0.0001	0.052	0.1628	710
C-reactive protein, mg/dL	1,32 (±2,77)	0.010	0.7662	-0.060	0.1150	-0.025	0.5208	0.004	0.9221	668
Heart ejection fraction (%)	61,35 (± 11,51)	-0.072	0.1883	0.107	0.0517	-0.046	0.4071	0.093	0.0901	331
Brain Parenchymal Fraction	0,95 (±0,02)	0.229	<0.0001	-0.068	0.3213	0.231	<0.0001	-0.086	0.2144	212

Table shows the set of variables that could not be imputed because we had information of them only in a subset of our cohort (last column, N of individuals with information for the variable) and thus were not used for the training. First column represents the distribution of these variables in the sample, presented as mean (±SD) or median (IQR). We present the correlation of each variable with the predictions and residuals from both best performing models in the test dataset (EN and MLP). Values represent the Pearson or Spearman's correlation coefficient (r), according to the distribution of each variable, and *p*-values.

Keywords: EN, elastic net regression; IQR, interquartile range; MLP, multilayer perceptron; SD, standard deviation.

Supplementary table S6: Correlation between best performing models' residuals and CpG β values included in Hannum's epigenetic clock

	EN Residuals		MLP Residuals	
**CpG**	**r**	***p*-value**	**r**	***p*-value**
cg00481951	0.293	<0.0001	0.295	<0.0001
cg00486113	-0.138	<0.0001	-0.136	<0.0001
cg00748589	0.14	<0.0001	0.142	<0.0001
cg01528542	-0.056	0.0859	-0.062	0.0587
cg02046143	-0.079	0.0151	-0.088	0.0067
cg02085953	-0.161	<0.0001	-0.166	<0.0001
cg02867102	-0.119	<0.0001	-0.122	<0.0001
cg03032497	0.276	<0.0001	0.274	<0.0001
cg03399905	0.266	<0.0001	0.269	<0.0001
cg03473532	-0.174	<0.0001	-0.184	<0.0001
cg03607117	0.228	<0.0001	0.225	<0.0001
cg04416734	-0.1	0.0022	-0.11	<0.0001
cg04474832	-0.311	<0.0001	-0.306	<0.0001
cg04875128	0.235	<0.0001	0.244	<0.0001
cg04940570	0.222	<0.0001	0.221	<0.0001
cg05442902	-0.132	<0.0001	-0.137	<0.0001
cg06419846	0.124	<0.0001	0.125	<0.0001
cg06493994	0.177	<0.0001	0.179	<0.0001
cg06639320	0.424	<0.0001	0.419	<0.0001
cg06685111	-0.161	<0.0001	-0.171	<0.0001
cg06874016	-0.255	<0.0001	-0.269	<0.0001
cg07082267	-0.253	<0.0001	-0.254	<0.0001
cg07547549	0.27	<0.0001	0.283	<0.0001
cg07553761	0.452	<0.0001	0.443	<0.0001
cg07583137	-0.222	<0.0001	-0.234	<0.0001
cg07955995	0.213	<0.0001	0.207	<0.0001
cg08097417	0.171	<0.0001	0.166	<0.0001
cg08234504	-0.194	<0.0001	-0.203	<0.0001
cg08415592	-0.343	<0.0001	-0.336	<0.0001
cg08540945	0.178	<0.0001	0.186	<0.0001
cg09809672	-0.13	<0.0001	-0.137	<0.0001
cg10501210	-0.08	0.0137	-0.096	0.0030
cg11067179	-0.003	0.9192	0.007	0.8368
cg14556683	0.164	<0.0001	0.162	<0.0001
cg14692377	0.35	<0.0001	0.351	<0.0001
cg16054275	-0.214	<0.0001	-0.222	<0.0001
cg16419235	0.212	<0.0001	0.207	<0.0001
cg16867657	0.245	<0.0001	0.256	<0.0001
cg19283806	-0.106	0.0011	-0.121	<0.0001
cg19722847	-0.051	0.1214	-0.049	0.1320

cg19935065	0.186	<0.0001	0.191	<0.0001
cg20052760	-0.195	<0.0001	-0.197	<0.0001
cg20426994	0.239	<0.0001	0.238	<0.0001
cg20822990	-0.29	<0.0001	-0.293	<0.0001
cg22016779	-0.188	<0.0001	-0.202	<0.0001
cg22158769	0.292	<0.0001	0.286	<0.0001
cg22213242	0.058	0.0762	0.06	0.0656
cg22285878	0.181	<0.0001	0.168	<0.0001
cg22454769	0.233	<0.0001	0.233	<0.0001
cg22512670	-0.231	<0.0001	-0.231	<0.0001
cg22736354	0.253	<0.0001	0.258	<0.0001
cg22796704	-0.18	<0.0001	-0.182	<0.0001
cg23091758	0.28	<0.0001	0.278	<0.0001
cg23500537	0.312	<0.0001	0.306	<0.0001
cg23606718	0.165	<0.0001	0.167	<0.0001
cg23744638	-0.074	0.0233	-0.085	0.0094
cg25410668	0.116	<0.0001	0.123	<0.0001
cg25478614	0.197	<0.0001	0.202	<0.0001

This table shows the correlation between best performing model'sresiduals (EN and MLP) with CpGs β-values included in Hannum's clock. Values represent the Pearson or Spearman's correlation coefficient's, depending on the distribution of each variable, and *p*-values.

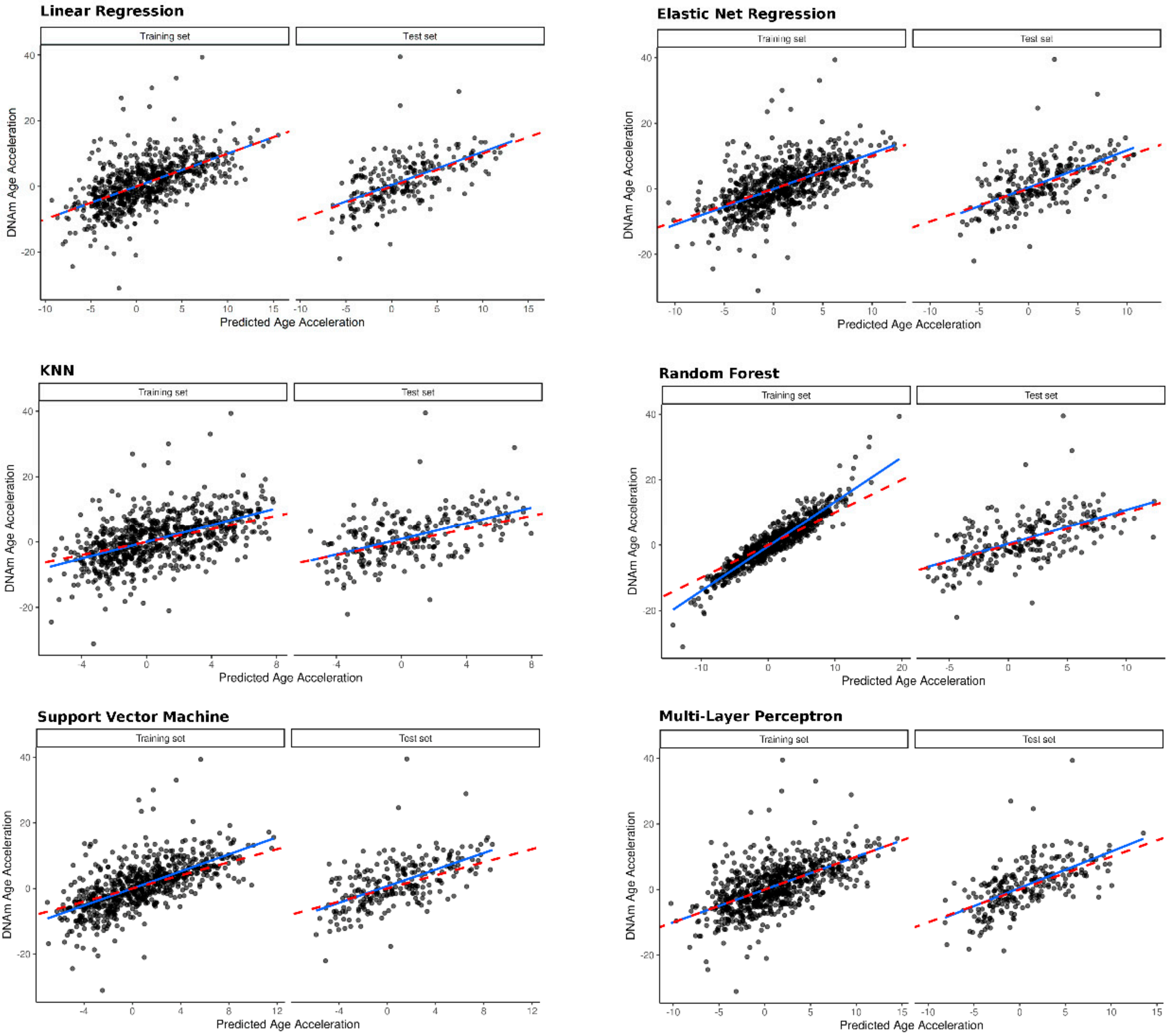

Supplementary figure S1: Correlation between predicted and actual Aging-A values for each model. We display the correlation between predicted and actual Aging-A values in both training and test datasets. Red dashed line shows the perfect adjustment, and the blue line shows the model linear trend.
